# APCAD Part 2: A Novel Method for Detection of Meiotic Aneuploidy in Preimplantation Embryos

**DOI:** 10.3390/genes16020115

**Published:** 2025-01-21

**Authors:** Pieter Verdyck, Veerle Berckmoes, Elia Fernandez Gallardo, Kathelijn Keymolen, Catharina Olsen, Martine De Rycke

**Affiliations:** 1 Research Group Genetics, Reproduction and Development (GRAD), Centrum Medische Genetica, Vrije Universiteit Brussel (VUB), Universitair Ziekenhuis Brussel (UZ Brussel), Laarbeeklaan 101, 1090 Brussels, Belgium; 2 Brussels Interuniversity Genomics High Throughput Core (BRIGHTcore), VUB-ULB, Laarbeeklaan 101, 1090 Brussels, Belgium; 3 Interuniversity Institute of Bioinformatics in Brussels, 1050 Unversité Libre de Bruxelles-Vrije Universiteit, 1090 Brussels, Belgium

**Keywords:** PGT-A, PGT-AO, meiotic, aneuploidy, method, APCAD

## Abstract

**Background/Objectives:** Preimplantation genetic testing methods to detect aneuploidy (PGT-A) based on genomewide single nucleotide polymorphism (SNP) data were scarce and did not meet our needs. **Methods:** Hence, we developed a novel method for this purpose. After the raw B-allele frequency (rBAF) values of Single Nucleotide Polymorfisms (SNPs) are obtained from a sample of interest with SNP array, the BAF values for specific categories of SNPs (cBAF) are visualized separately. **Results:** The analysis of the cBAF, rBAF and Log_2_R profiles enables to distinguish all common types of chromosomal abnormalities without haplotyping. This was demonstrated by reanalyzing data from 359 embryos which had previously been analyzed with Karyomapping. We identified additional underrepresented maternal haplotypes in five samples that we could not detect with Karyomapping. In addition, we identified all chromosomes with meiotic-origin copy number gains (both parental homolog (BPH)) (n = 70) and all chromosomes with a non-mosaic copy number loss larger than 5 Mb (n = 93) that had been detected with Karyomapping. **Conclusions:** We conclude that the proposed method can be used to reliably detect meiotic-origin aneuploidy without haplotyping and, hence without the need for a phasing reference.

## 1. Introduction

Karyomapping (Vitrolife, formerly Illumina) allows reliable preimplantation genetic testing for monogenic disorders (PGT-M) in biopsy samples obtained from in vitro fertilized (IVF) embryos [1,2]. Karyomapping can also be used for preimplantation testing for structural rearrangements (PGT-SR) and has the benefit that normal segregations of translocations and inversions can be distinguished from balanced segregations [3]. The availability of genomewide SNP data also enables aneuploidy detection, by visualizing the haplotypes, the copy number (Log_2_R) and raw B-allele frequency (rBAF) values [4,5,6]. The B-allele frequency (BAF) reflects the proportion of B-alleles (G and C nucleotides in Karyomapping) present in the sample at the position of the SNP. If both haplotypes from one parent are observed for a certain chromosome, a meiotic chromosome anomaly is evident. These anomalies such as trisomies, duplications and uniparental heterodisomies can be collectively referred to as ’both parental homolog (BPH) anomalies [6,7]. Monosomies and deletions are detected by the absence of both haplotypes from one parent. On the other hand, some types of anomalies will remain undetected by haplotyping, for example, if a parent transmitted two identical copies of a chromosome or chromosome segment. The resulting anomalies, mainly trisomies and duplications, are collectively called ’single parental homolog’ (SPH) anomalies [6,7]. These SPH and mosaic anomalies can only be detected with Karyomapping based on the rBAF and/or Log_2_R profiles. However, this may be cumbersome, especially in samples of lower quality. In addition, depending on the reference used with karyomapping, the maternal or paternal haplotypes may not be available. For example, when using a paternal grandparent as reference, the maternal haplotypes are not determined in the embryos, potentially masking a maternal BPH trisomy.

To overcome these issues, we developed a method leveraging the use of the available SNP allele frequency data. We previously showed that visualization and calculations based on a selection of SNPs, termed ‘Analysis of Parental Contribution for Aneuploidy Detection’ (APCAD) SNPs, provide a useful intuitive tool to identify aneuploidy in blastocyst biopsies [8]. They are useful to distinguish mosaic from complete aneuploidy and estimate the proportion of aneuploid cells independent of copy number/Log_2_R. By selecting additional categories of SNPs and displaying these in genome-wide plots, we aimed to improve our method further so it can reliably detect all common types of aneuploidy and distinguish BPH and SPH trisomies. This distinction is important, given that BPH trisomies are meiotic in origin i.e., present in the gametes, and the resulting embryo is expected to be uniformly aneuploid. In contrast, SPH trisomies are expected to be predominantly mitotic in origin, especially in combination with an intermediate copy number, even though a meiotic segregation error in meiosis II without recombination cannot be excluded [7]. We previously showed that SPH trisomies are more frequently diagnosed mosaic by APCAD compared to BPH trisomies, as expected [8].

Preimplantation genetic testing for aneuploidy (PGT-A) has been dominated to date by array comparative genomic hybridization and subsequently by shallow whole genome sequencing (sWGS), technologies that can only be used to compare the relative amount of DNA between chromosomes in a sample. In other words, embryos are diagnosed as euploid when all chromosomes are equally represented. Conversely, when the number of reads increases by 1.5-fold or decreases to 0.5× for a chromosome, the presence of a full trisomy or full monosomy is concluded respectively. When a copy number between normal and fully aneuploid is detected, a so called ’intermediate’ copy number, mosaicism is suspected: the mixture of euploid and aneuploid cells in the biopsy.

The transfer of embryos with intermediate copy numbers detected by sWGS has been shown to lead to healthy offspring. Large cohort studies have shown that the transfer of these embryos is safe, even though they have a lower implantation potential and a higher risk for miscarriage [9,10]. Others, via non-selection studies, claim that this is rather due to selection bias: couples with poor prognosis choose to have a mosaic embryo replaced, negatively influencing the outcome, concluding that mosaic embryos should be treated equal to euploid embryos [11].

Interestingly, embryos with high-grade mosaicism in the trophectoderm biopsy have a worse outcome compared to low level mosaic [9], and the corresponding inner cell mass is more frequently abnormal [11]. In addition, a recent study has shown that embryos with intermediate copy numbers can harbour a meiotic-origin trisomy [12]. Therefore we can hypothesized that biopsies with a (high-grade) intermediate copynumber are sometimes obtained from embryos with a meiotic-origin trisomy. Transfer of such embryos with a meiotic trisomy is to be avoided given that the inner cell mass is expected to be uniformly aneuploid. Hence, transfer would almost invariably lead to failed implantation, miscarriage or even an aneuploid child. Therefore, aneuploidy screening that includes detection of aneuploidy origin, recently coined PGT-AO by others [13], would improve the accuracy of PGT-A, leading to better selection of embryos without meiotic aneuploidies than the current standard practice that only relies on detection of copy number.

## 2. Materials and Methods

### 2.1. IVF Treatment and Embryo Manipulations

IVF treatment including stimulation, oocyte collection, ICSI, embryo culture, trophectoderm biopsy and vitrification were previously described [14].

### 2.2. Genomic DNA and Embryos

For this retrospective analysis, we selected embryos subjected to trophectoderm biopsy in our center between 1 September 2015 and 31 December 2017 diagnosed with Karyomapping as not genetically transferable for PGT-M and/or PGT-SR. A total of 359 embryos with SNP data of sufficient quality (call rate >85%, ≤1% miscall rate) and written informed consent were finally included. The majority of the embryos were diagnosed for a PGT-M indication (n = 341) and a minority for PGT-SR or a combination of PGT-SR and PGT-M (n = 18). The data from these embryos was reanalyzed and cBAF profiles were generated. Given that the SNP data was readily available after preimplantation genetic testing with SNP array (Karyomapping), no additional intervention on the patient or embryo was required for the current study. The study was approved by the local ethical committee under number B.U.N. 143201731745. The cohort is identical to the cohort previously published in Verdyck et al., 2022 [8]. Three complete embryos that were not genetically transferable were collected and reanalyzed after informed consent.

### 2.3. WGA and SNP Array

Karyomapping version 1 workflow was performed, including whole genome amplification (WGA) and SNP array analysis, according to the manufacturer’s recommendations (Illumina, San Diego, CA, USA/Vitrolife, Gothenburg, Sweden).

## 3. Results

### 3.1. Improved Visualization Using cBAF Profiles

We defined four distinct SNP categories based on the genotypes of the parents for improved aneuploidy detection (Table 1). We visualized the BAF obtained from the trophectoderm sample for each category separately (cBAF).

**Figure 1:**
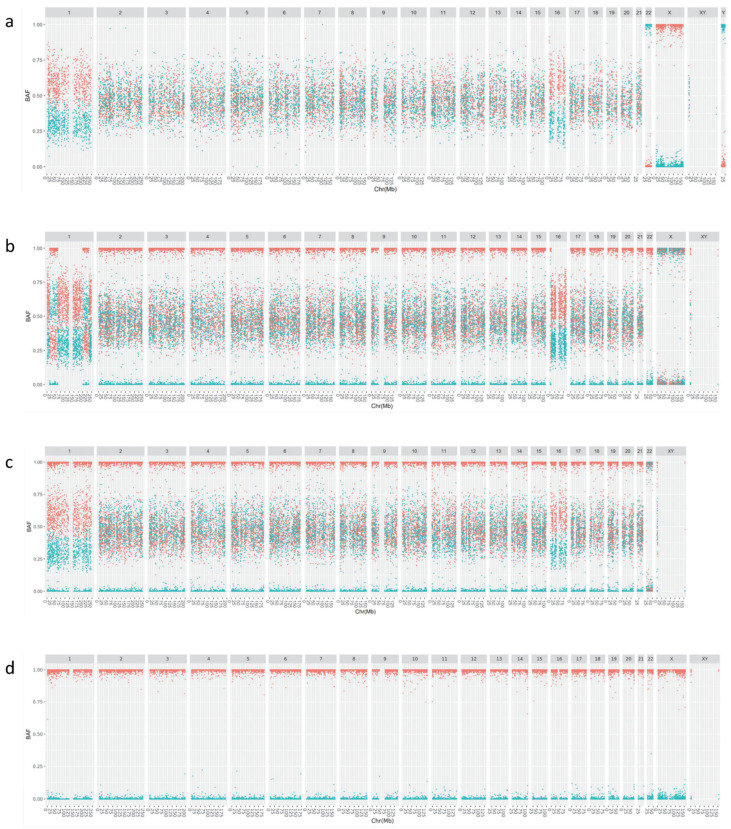

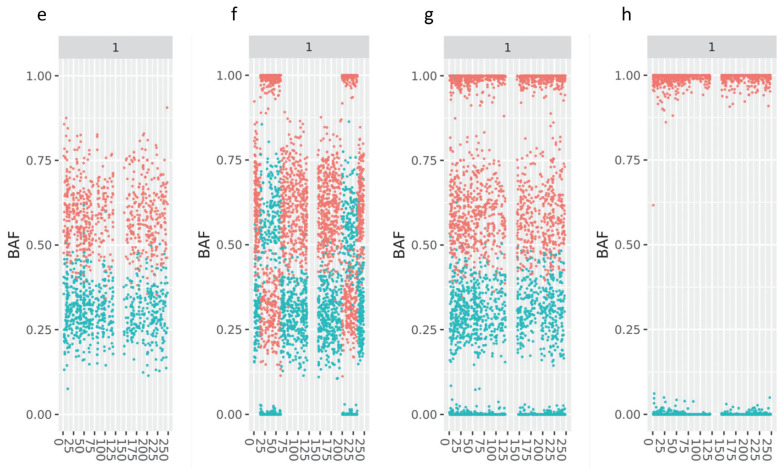
Categorized BAF plots from a day 6 trophectoderm biopsy diagnosed as 47,XY,+1,+16,−22. For each SNP category (**a**–**h**) the BAF values of the SNPs are shown (Y-axis) according to their genomic location (X-axis; chromosome and chromosome position in Mb). Without the use of reference for phasing, we can observe the presence of a maternal monosomy 22 and a maternal meiotic trisomy 1 and 16. (**a**) Category 1 SNPs: SNPs for which both parents have a homozygous genotype for a different allele. These SNPs should be heterozygous for chromosomes with normal disomy. For SNPs of subcategory 1A (blue), the BAF corresponds to the proportion of alleles of paternal origin (paternal BB and maternal AA genotype) while the BAF of subcategory 1B SNPs (red) corresponds to the proportion of alleles of maternal origin (paternal AA and maternal BB genotype). SNPs on the Y chromosome are added with a BB (to subcategory 1A) or AA (to subcategory 1B) call from the father and a very low signal intensity (log_2_R < −4) or no call from the mother. (**b**) Category 2 SNPs: maternal informative SNPs. Subcategory 2A (paternal BB and maternal AB call; red) and subcategory 2B (paternal AA and maternal AB call; blue). Note the striking pattern for the maternal origin meiotic trisomy of chromosomes 1 and 16. (**c**) Category 3 SNPs: paternal informative SNPs. Subcategory 3A (paternal AB and maternal BB call; red) and subcategory 3B (paternal AB and maternal AA call; blue). (**d**) BAF of category 4 SNPs: obligate homozygous SNPs. Subcategory 4A (paternal BB and maternal BB call; red) and subcategory 4B (paternal AA and maternal AA call; blue). These SNPs are used for detection of contamination and inspection of the noise for homozygous SNPs. (**e**)**:** Detail of category 1 SNPs of chromosome 1 shown in panel (**a**). (**f**): Detail of category 2 SNPs of chromosome 1 shown in panel (**b**). (**g**): Detail of category 3 SNPs of chromosome 1 shown in panel (**c**) (**h**): Detail of category 4 SNPs of chromosome 1 shown in panel (**d**).

Figure 1 shows cBAF profiles for anembryo with a 47,XY,+1,+16,−22 karyotype. A view of the Log_2_R and raw BAF (rBAF) from the Bluefuse software is shown in Figure S1 for comparison.

The first category consists of SNPs for which both parents show a homozygous genotype for a different allele (Figure 1a and Table 1). These SNPs are expected to be heterozygous for all chromosomes with normal disomy, We previously named these SNPs the APCAD SNPs [8]. Note that the BAF for these selected SNPs reflect the proportion of alleles of paternal origin (subcategory 1A; blue) and of maternal origin (subcategory 1B; red). In the example (Figure 1a,e) a deviating maternal and paternal contribution can be observed for chromosome 22 (no maternal copy), chromosomes 1 and 16 (maternal origin trisomy), X and Y (male embryo).

We added three additional categories of SNPs to the category described above. The second and third categories of SNPs are the unphased maternal and paternal informative SNPs, respectively i.e., heterozygous in the mother, homozygous in the father (Figure 1b,f) and heterozygous in the father, homozygous in the mother (Figure 1c,g). The fourth category consists of SNPs for which both parents have a homozygous genotype for the same allele (both AA or both BB). The embryo also should have a homozygous genotype for these SNPs (Figure 1d,h). This category is useful for quality control as it allows the analysis of the BAF distribution of homozygous SNPs and the detection of external origin contamination. In analogy with the category 1 SNPs, we also defined subcategories for category 2, 3 and 4 SNPs based on the parental genotypes (Table 1).

**Figure 2:**
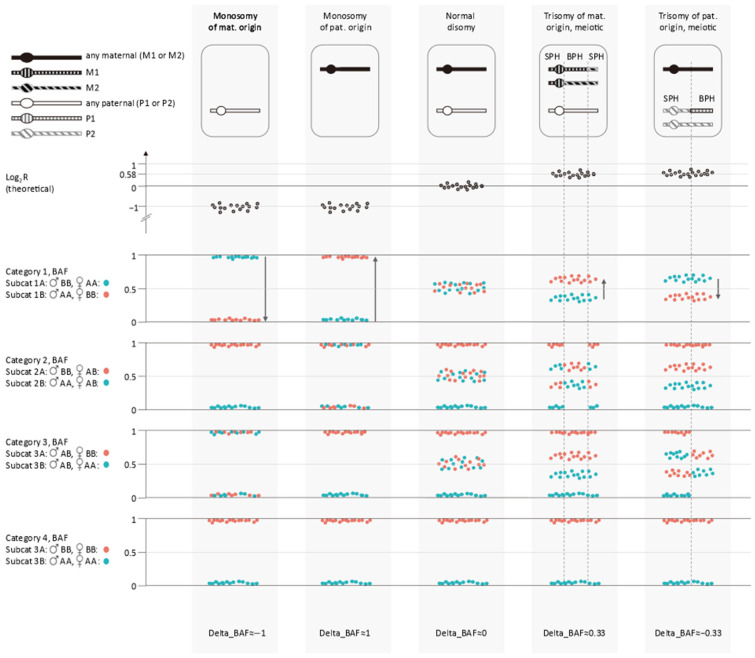
Schematic overview of expected Log_2_R and BAF profiles for disomy, monosomy and trisomy. For clarity the number of BAF values is greatly reduced. The ’delta_BAF’ value is calculated based on the difference in mean BAF of both subcategories of category 1 SNPs (black arrows). Mind that all mentioned chromosomal anomalies can be discriminated using a combination of Log_2_R values and the patterns observed for the BAF of category 1, 2 and 3 SNPs. Even though no haplotyping is used, meiotic and mitotic origin trisomies can be discriminated. ‘M1’: maternal haplotype 1, ‘M2’: maternal haplotype 2, ‘P1’: paternal haplotype 1, ‘P2’: paternal haplotype 2. ‘♂’: paternal genotype, ‘♀’: maternal genotype, ‘IBD’: region that is identical by descent with female and male partner sharing a haplotype, ‘SPH’: single parental homolog present, ‘BPH’: both parental homologs present. Mat.: maternal; pat.: paternal.

We refer to the cBAF, rBAF and log_2_R profiles together as the APCAD profiles. Inspection of the APCAD profiles allows aneuploidy detection and detection of copy number gains of meiotic origin. In the example (Figure 1), even without a phasing reference, we can conclude that the monosomy 22, trisomy 1 and trisomy 16 are of maternal origin. Recombinations in the chromosomes with a maternal meiotic-origin trisomy create a distinctive pattern (Figure 1b,f). Regions without homozygous SNPs (two different maternal haplotypes present) alternate with regions that do contain homozygous SNPs (two identical maternal copies). We can conclude that the observed trisomies in the example originate from meiosis I, based on the absence of homozygous SNPs around the centromeres of chromosomes 1 and 16. Figure 2 and Figure S2 depict a schematic overview of the different expected patterns for disomy and the most common whole chromosome abnormalities. Segmental abnormalities should exhibit the same patterns, but only for part of the chromosome.

### 3.2. Retrospective Analysis Using APCAD Profiles

The SNP array data from 359 embryos were retrospectively reanalyzed by APCAD (cBAF, rBAF and Log_2_R) for the presence of meiotic (BPH) copy number gains or non-mosaic chromosome copy number losses larger than 5 Mb, without accessing the available haplotype information. Afterwards, we compared the APCAD result interpretation with the findings obtained with Karyomapping (haplotype profiles, rBAF and Log_2_R). We excluded mosaic copy number losses and SPH copy number gains from the comparison due to the low sensitivity of Karyomapping in detecting them.

In terms of genome-wide abnormalities, we identified an abnormal ploidy with both methods in five embryos. Two haploid embryos only carried a chromosome set of maternal origin, while three triploid embryos showed an additional maternal chromosome complement. In four other embryos, we detected the genomewide presence of an additional underrepresented maternal haplotype with APCAD that had not been detected by Karyomapping (example shown in Figure S3). By analyzing short tandem repeat (STR) markers, we confirmed the presence of the second maternal allele in all four samples, with a relatively low peak height, in a subset of STR markers (not shown). In three of the biopsies, the BPH anomaly was present in a seemingly segmented manner on all chromosomes, indicative of a second polar body contamination (Figure S3). In the fourth biopsy the maternal BPH anomaly was present apparently uniformly across all chromosomes (not shown). Reanalysis of two of the complete embryos (2/4), showed absence of the underrepresented maternal haplotype in the remainder of the embryo.

In the remaining 350 embryos, both methods identified 70 chromosomes with a BPH copy number gain and 93 chromosomes with a copy number loss larger than 5 Mb, albeit with a different observed copy number in two occurrences (Table 2 and Table 3). A segmental BPH copy number gain with one additional copy (+1; duplication) with Karyomapping was interpreted as a segmental BPH copy number gain with 2 additional copies (+2; triplication) by analysis of APCAD profiles. For one chromosome, a monosomy was identified with Karyomapping while a 49 Mb deletion was detected with APCAD, together with a high-grade mosaic loss of the remainder of the chromosome (Table 3).

For five chromosomes a different interpretation was obtained after analysis of APCAD profiles compared to Karyomapping (Table 3). Apart from the two chromosomes with a different non-mosaic copy number abnormality described above, there were three chromosomes interpreted as normal disomy (n = 1) or mosaic (n = 2) after Karyomapping that were diagnosed with a non-mosaic copynumber abnormality using APCAD. Two mosaic monosomies detected with Karyomapping were shown with APCAD to harbour a full paternal deletion estimated to be present in all cells, together with a high-grade mosaic loss of the remainder of the chromosome. For the third chromosome, a relatively small (~6 Mb, Figure S4) BPH anomaly was observed in low grade on chromosome 1q23.3q24.2. Analysis with STR markers confirmed this finding. Reanalysis of the complete embryo with APCAD confirmed the presence of a BPH anomaly, again in low grade, in the remainder of the embryo, but the involved segment was considerably larger (~50 Mb, Figure S4).

Summarizing the results, we identified all 163 chromosomes with a BPH anomaly or complete copy number loss larger than 5 Mb with APCAD that were observed by Karyomapping as the reference method. Therefore, we calculated the sensitivity of APCAD per chromosome for this type of abnormalities as 100% [163 true positives/(163 true positives + 0 false negatives)]. We observed a BPH anomaly or complete copy number loss larger than 5 Mb for three chromosomes with APCAD that we had not detected with Karyomapping. With Karyomapping as gold standard, we calculate the specificity of APCAD per chromosome as 99.96% [8234 true negatives/(8234 true negatives + 3 false positives)]. However, given that in at least of 1 of 3 discordances the APCAD result was confirmed using STR markers, the specificity of APCAD for the tested types of chromosomal anomalies could be even closer to 100%.

Of note, deletions smaller than 5 Mb were not in the scope of this comparison, given that we considered them insufficiently detectable by Karyomapping. However, in embryos from seven couples, we observed nine smaller deletions, sized between 126 and 626 kB. The APCAD profiles showed that six deletions were of paternal origin and three of maternal origin. They were all inherited familial deletions that could be confirmed using the available rBAF and/or Log_2_R profiles obtained from the parent indicated by APCAD. The deletions were classified as benign (n = 7) or likely benign (n = 2). See Figure S5 for an example.

## 4. Discussion

We have developed a novel SNP based approach for detection of aneuploidy in embryos based on different SNP (sub)categories, defined by the SNP genotype combinations from the parents. The BAF of these selected SNPs is visualized and complement the raw BAF and Log_2_R profiles. Based on these profiles, all common types of chromosomal anomalies were detected, without the requirement of a reference sample for haplotyping. In addition, the type of anomaly (e.g., maternal versus paternal origin, single or both parental homologs present, meiosis I versus meiosis II trisomy) was identified independent of the availability of reference samples. This was shown by reanalyzing data from 359 embryos which had previously been analyzed with Karyomapping.

For determining the accuracy of APCAD, a subset of 350 embryos without genomewide chromosomal abnormalities (haploid, triploid or genome wide underrepresented haplotypes) was analyzed, with Karyomapping as a gold standard. All chromosomes (n = 70) with BPH anomalies and all chromosomes with a full copy number losses larger than 5 Mb (n = 93) that had been identified with Karyomapping were discerned, implying a 100% sensitivity. Five chromosomes yielded a discordant result. Given that two chromosomes were identified as abnormal by both methods, they are considered true positives, even though there were differences in conclusions about the type of abnormality. For three other chromosomes diagnosed as mosaic monosomy or normal disomy by Karyomapping (n = 8237), we observed a BPH anomaly (n = 1) or non-mosaic deletion (n = 2), implying a 99.96% specificity per chromosome when taking Karyomapping as reference.

However, we are confident that the APCAD method is more accurate than Karyomapping as it makes use of continuous BAF values rather than discrete genotype calls. If a haplotype is underrepresented, the allele frequency values may not reach the threshold for heterozygous genotype calling in Karyomapping and the haplotype will remain undetected. By using the continuous allele frequency values (BAF) with APCAD, we were able to detect the presence of such underrepresented haplotypes. In this way, we identified four samples (4/354; 1.1%) with an additional underrepresented genomewide maternal haplotype next to a well-represented maternal and paternal haplotype. This finding was confirmed by analysis of STR markers. Given that the additional haplotype was always of maternal origin, and that it was not present when analyzing the remainder of the embryo (2/4 tested), we consider it most likely that the presence of an underrepresented genomewide maternal haplotype is due to DNA fragments from a (degenerating) polar body as previously reported [15]. The seemingly segmented pattern with lack of BPH anomalies around centromeres is indicative for a second polar body in three out of four occurrences. The fourth occurrence could be compatible with cumulus cell or first polar body contamination.

For one chromosome, a low-grade BPH anomaly was observed for a short (~6 Mb) segment. Analysis of the remainder of the embryo confirmed the presence of a low-grade BPH anomaly at the same genomic location, even though larger in size (~50 Mb). The involved mechanism and clinical significance remains elusive.

Similarly, APCAD allowed better distinction of high-grade mosaic copy number loss from full (non-mosaic) copy number loss. For three chromosomes, a full (non-mosaic) paternal origin deletion was observed, together with a high-grade mosaic copy number loss of the remainder of the chromosome using APCAD. In two of these, a high-grade mosaic monosomy was diagnosed with Karyomapping. Such full deletion is potentially viable and could cause miscarriage or the abnormal development of the fetus if transferred. When embryos with high-grade mosaic monosomy are considered for transfer, caution needs to be taken that full deletions are absent on this chromosome.

The analysis of APCAD plots is convenient as the chromosomal abnormalities such as monosomies, deletions and meiotic-origin trisomies clearly stand out visually when analyzing the genome wide profiles. Also, the allele frequency values are less subject to amplification bias compared to estimated copy number values. Especially in GC rich regions (e.g., at telomeres, centromeres, chromosomes 19 and 22) detection of copy number abnormalities can be challenging after WGA. As we compare SNP AF values within a sample, and both alleles are subject to the same (GC) amplification bias, this issue is largely overcome. In addition, because APCAD profiles allow detection of aneuploidy in a fundamentally different way compared to copy number, aneuploidy observed with one of both can be confirmed with the other. E.g., when a deletion is suspected based on copy number, the finding can be confirmed by analysis of the APCAD profiles.

The APCAD method allows aneuploidy detection from SNP data using DNA from the prospective parents only, avoiding the need for a phasing reference. This was a unique feature at the time of its development compared to other available SNP based methods, as they rely on haplotyping [2,16], even though a recent update of the haplaritmisis method allows a trio analysis without reference for PGT-AO [13]. Acquiring a reference sample (i.e., sibling or grandparents) may represent a burden as the prospective parents do not always wish to disclose the need for PGT and/or IVF to their relatives. In other cases, the (prospective) grandparents are deceased or contact has been lost. Also, in situations where only one grandparental reference (e.g., maternal side) is used, APCAD allows aneuploidy detection without requesting a sample from another grandparent (e.g., paternal side).

A limitation of the APCAD method, like haplotyping methods, is that in case of consanguinity aneuploidy may be more difficult to detect. If both partners share a haplotype that is identical by descent, the cBAF profiles may be more difficult to interpret for this chromosome segment as there will be no category 1 SNPs available in this region since the parents will never have SNPs with a different homozygous genotype (maternal AA and paternal BB or vice versa). Category 2 and 3 SNPs will all be heterozygous or homozygous depending on the maternal and paternal haplotype that was transmitted (Figure S2). Note that due to loss of homozygous SNPs for categories 2 and 3, cBAF profiles for a normal disomy may be similar (but not identical) to the cBAF profile for a BPH anomaly.

Further investigation is required on the APCAD method regarding its resolution. APCAD allows detection of SPH and BPH segmental copy number gains and losses. More specifically, thirty deletions with a size between 10 and 123 Mb were detected. Deletions smaller than 5 Mb, even smaller than 0.5 Mb, were also detected using APCAD, but due to lack of comparison, sensitivity and specificity for such small deletions could not be determined during this study. Given that only few and predominantly larger duplications were detected in the current dataset, also the sensitivity for detection of duplications is currently unknown.

We showed that the genome-wide plots of categorized SNPs with APCAD allow reliable aneuploidy detection on multiple displacement amplified (MDA) trophectoderm samples from preimplantation embryos. However, it is a generic method that can be applied on all data with good quality allele frequency values, including data obtained with high depth whole genome sequencing and data obtained from PCR-based WGA material (e.g., Sureplex/Picoplex) or genomic DNA (not shown).

To date, PGT-A is predominantly performed by trophectoderm biopsy, PCR based whole genome amplification and shallow whole genome sequencing (sWGS). Even though the technology is widely adopted and has proven useful, it is a rather simple method that has several limitations. First, not all types of abnormalities can be detected. Abnormal ploidy (haploidy, triploidy) and uniparental disomy (UPD) remain undetected. Second, (high level) contamination could remain undetected which can lead to aneuploid embryos being diagnosed as euploid or mosaic. Also, a meiotic abnormality such as a meiotic trisomy could be masked by a coincidental mitotic loss of the same chromosome in the trophectoderm. Given that transfer of aneuploid embryos has a futile prognosis [17,18] and detection of mosaic errors of mitotic origin has limited clinical validity, Janssen et al. 2024 [13] have suggested the implementation of PGT for aneuploidy origin (PGT-AO). With the current method, such PGT-AO can be implemented. Lastly, PGT-AO with APCAD offers benefit of verifying that the correct gametes (from the correct donor or partner) were used for fertilization.

## 5. Patents

The method described in this study is subject of patent application WO2021180722.

## Figures and Tables

**Table 1:** The used categories and subcategories of SNPs.

Categories	Paternal Genotype Call	Maternal Genotype Call	Expected Genotype Sample *	Remark
Category 1				
Subcategory 1A	BB	AA	AB	
	BB	NC	NC or BB	Only for SNPs on Y chr.
Subcategory 1B	AA	BB	AB	
	AA	NC	NC or AA	Only for SNPs on Y chr.
Category 2				
Subcategory 2A	BB	AB	AB or BB	Not phased
Subcategory 2B	AA	AB	AA or AB	Not phased
Category 3				
Subcategory 3A	AB	BB	AB or BB	Not phased
Subcategory 3B	AB	AA	AA or AB	Not phased
Category 4				
Subcategory 4A	BB	BB	BB	
Subcategory 4B	AA	AA	AA	

* SNP genotype calls are shown as AA, AB, BB or NC. “NC” indicates no call or very low signal intensity (log_2_R < −4). Hemizygous SNPs A/- and B/- are written as AA or BB respectively as hemizygosity and homozygosity cannot be distinguished.

**Table 2:** Summary of the concordant results per chromosome regarding chromosomal abnormalities with BPH copy number gains or full (non-mosaic) copy number loss, detected by analysis of Karyomapping and APCAD profiles.

Type of Anomaly	N of Mat. Origin	N of Pat Origin	Remark
De novo abnormalities (PGT-A)			
BPH trisomy autosome	56	1	
BPH trisomy XXY	3	2	
BPH segmental CN gain autosome	1	0	Sized 84 Mb
BPH segmental CN gain and deletion on autosome	1	0	Isochromosome 9q (deletion of 9p and BPH copy number gain of 9q)
Monosomy autosome	53	3	
Monosomy X	0	6	
Deletion on autosome	2	23	Maternal: sized 13 and 84 Mb. Paternal: sized 10, 14, 19, 25, 27, 30, 31, 32, 34, 40, 43, 47, 50, 51, 65, 65, 70, 78, 78, 81, 93, 103 and 123 Mb.
Deletion on X	1	0	Sized 58 Mb.
Inherited abnormalities (PGT-SR)			
BPH tetrasomy autosome	1	0	
BPH trisomy autosome	2	0	
BPH segmental CN gain autosome	2	0	Sized 58 and 77 Mb
Monosomy autosome	1	0	
Deletion on autosome	2	0	Sized 11 and 29 Mb

CN: copy number. BPH: both parental homolog. Pat: paternal origin, Mat: Maternal origin.

**Table 3:** Discordant results per chromosome regarding chromosomal abnormalities with BPH copy number gains or full (non-mosaic) copy number loss, detected by analysis of Karyomapping or APCAD profiles.

	Interpretation Karyomapping	Interpretation APCAD
1	BPH CN gain 17q21.34 to qter (2× mat)	BPH CN gain 17q21.34 to qter (3× mat) (~38 Mb)
2	Normal disomy	BPH CN gain 1q23.3 to q24.2 (2× mat; ~6 Mb), estimated mosaic
3	Mosaic monosomy chr10 in the majority of the analyzed cells	Pat deletion of 10q23.1 to 10q24.31 (~20 Mb) and mosaic CN loss of remainder of chr10 in the majority of the analyzed cells *
4	Mosaic monosomy chr3 in the majority of the analyzed cells	Pat deletion of pter to 3p24.2 (~25 Mb) and mosaic CN loss of remainder of chr3 in the majority of the analyzed cells *
5	Monosomy chr17 pat	Pat deletion of 17q12 to qter (~49 Mb) and mosaic CN loss of the remainder of chr17 in the majority of the analyzed cells *

* CN: copy number. BPH: both parental homologs. Pat: paternal origin, * Low paternal contribution observed for the remainder of the chromosome indicates a limited proportion of cells within the biopsy with a derivative chromosome and a majority of cells with a monosomy (no paternal copy).

## Data Availability

The data supporting the conclusions of this article will be made available by the authors on request. Due to privacy restrictions, this does not include raw SNP data from patients or embryos to prevent identification by comparison with public genetic databases.

## Supplementary Materials

The following supporting information can be downloaded at: https://www.mdpi.com/article/10.3390/genes16020115/s1, Figure S1. Screenshot of Bluefuse software for the same embryo with a 47,XY,+1,+16,−22 shown in Figure 1 for comparison with Figure 1; Figure S2: Schematic overview of expected Log_2_R and BAF profiles for other types of whole chromosome anomalies than shown in Figure 2; Figure S3. Trophectoderm sample with a genomewide underrepresented maternal haplotype that was not observed in the corresponding reanalyzed whole embryo; Figure S4. Profile of APCAD category 2 SNPs of a trophectoderm sample with a relatively short (~6 Mb) underrepresented maternal haplotype that was observed with APCAD but not Karyomapping; Figure S5. Example of a 0.4 Mb inherited deletion detected with APCAD but not with Karyomapping.
