# Supplementary material for: APCAD Part 2: A Novel Method for Detection of Meiotic Aneuploidy in Preimplantation Embryos
Source: Genes (Basel). 2025 Jan 21;16(2):115. doi: 10.3390/genes16020115 (PMC11854904; doi:10.3390/genes16020115)
Supplement: Supplementary file 1 [file genes-16-00115-s001.zip › Figure S2_250120.pptx]

## Slide 1
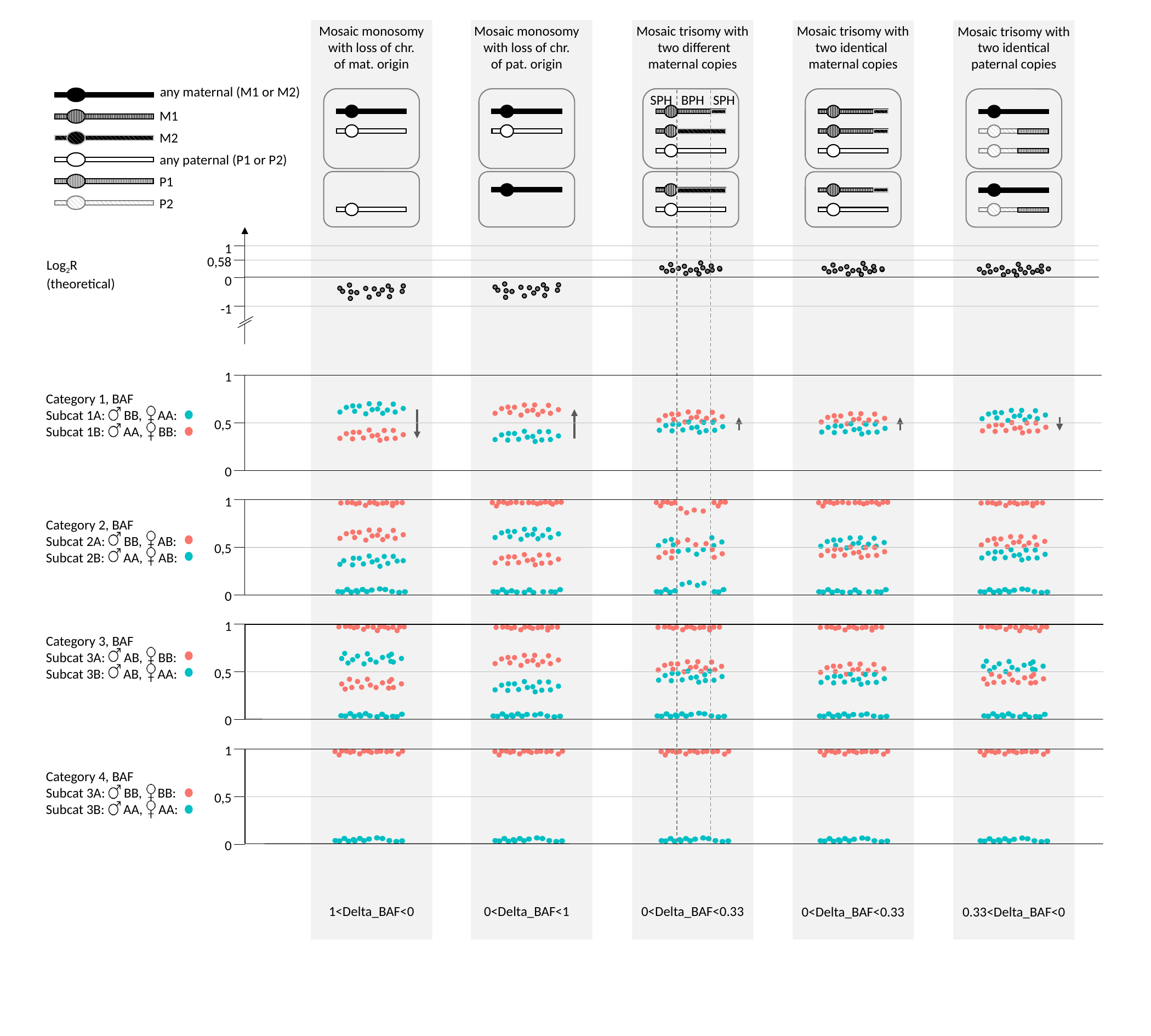

B
Mosaic monosomy
with loss of chr.
of mat. origin
Mosaic monosomy
with loss of chr.
of pat. origin
0<Delta_BAF<1
Mosaic trisomy with
 two different
maternal copies
Mosaic trisomy with
two identical
maternal copies
0<Delta_BAF<0.33
Mosaic trisomy with
two identical
paternal copies
any maternal (M1 or M2)
M1
M2
any paternal (P1 or P2)
P1
P2
SPH
BPH
SPH
1
0,58
0
-1
Log2R
(theoretical)
1
0,5
0
Category 1, BAF
Subcat 1A: BB, AA:
Subcat 1B: AA, BB:
1
0,5
0
Category 2, BAF
Subcat 2A: BB, AB:
Subcat 2B: AA, AB:
1
0,5
0
Category 3, BAF
Subcat 3A: AB, BB:
Subcat 3B: AB, AA:
1
0,5
0
Category 4, BAF
Subcat 3A: BB, BB:
Subcat 3B: AA, AA:
0<Delta_BAF<0.33

## Slide 2
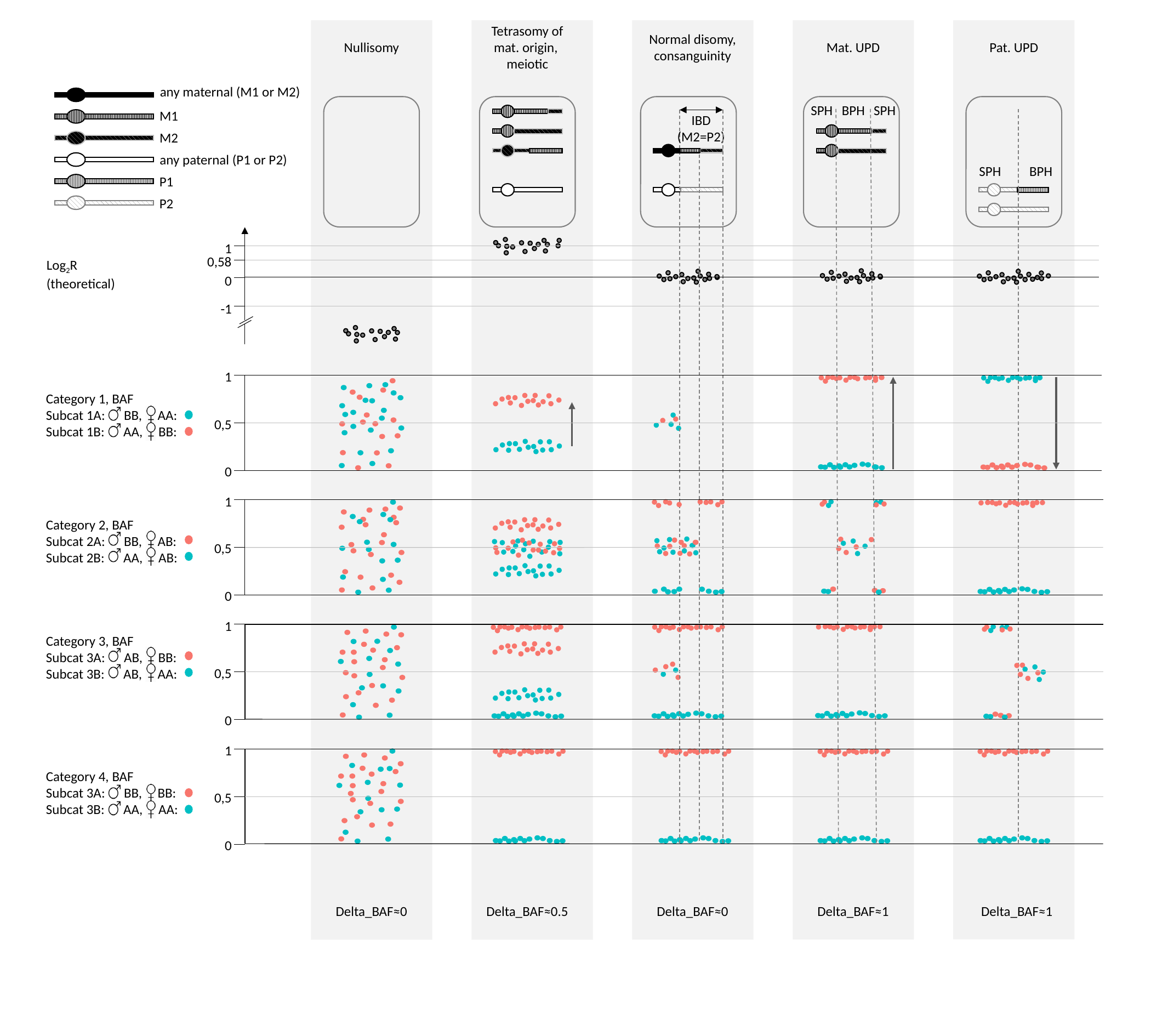

C
Tetrasomy of
mat. origin,
meiotic
Delta_BAF≈0.5
Normal disomy,
consanguinity
IBD
(M2=P2)
Delta_BAF≈0
Mat. UPD
SPH
BPH
SPH
Delta_BAF≈1
Pat. UPD
SPH
BPH
Nullisomy
Delta_BAF≈0
any maternal (M1 or M2)
M1
M2
any paternal (P1 or P2)
P1
P2
1
0,58
0
-1
Log2R
(theoretical)
1
0,5
0
Category 1, BAF
Subcat 1A: BB, AA:
Subcat 1B: AA, BB:
1
0,5
0
Category 2, BAF
Subcat 2A: BB, AB:
Subcat 2B: AA, AB:
1
0,5
0
Category 3, BAF
Subcat 3A: AB, BB:
Subcat 3B: AB, AA:
1
0,5
0
Category 4, BAF
Subcat 3A: BB, BB:
Subcat 3B: AA, AA:
