# Supplementary figures and images for: APCAD Part 2: A Novel Method for Detection of Meiotic Aneuploidy in Preimplantation Embryos
Source: Genes (Basel). 2025 Jan 21;16(2):115. doi: 10.3390/genes16020115 (PMC11854904; doi:10.3390/genes16020115)

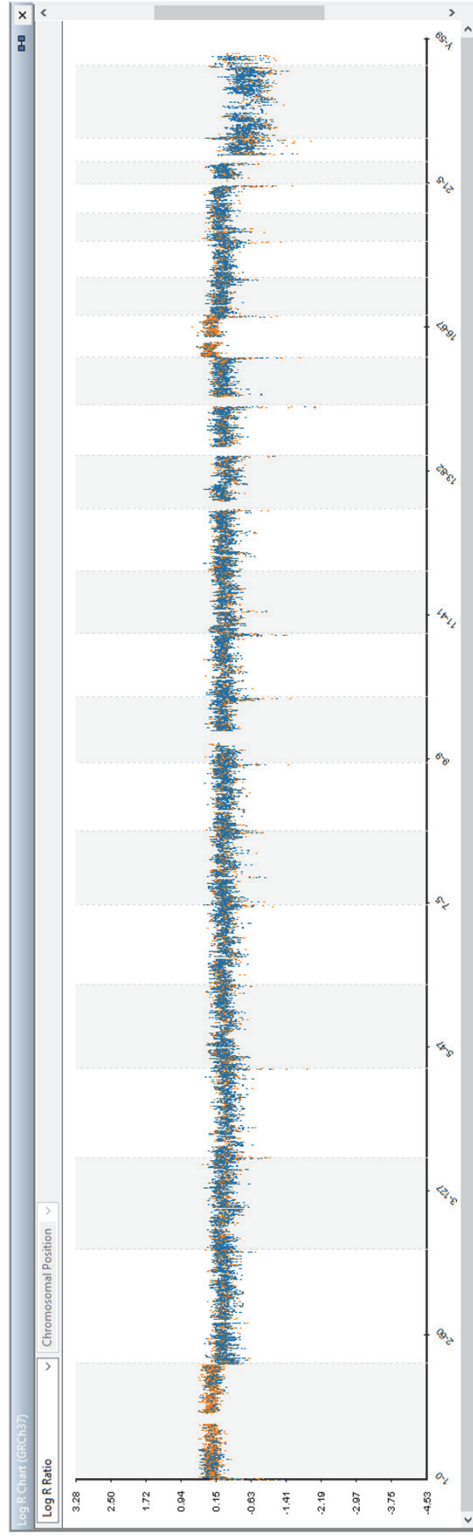

a

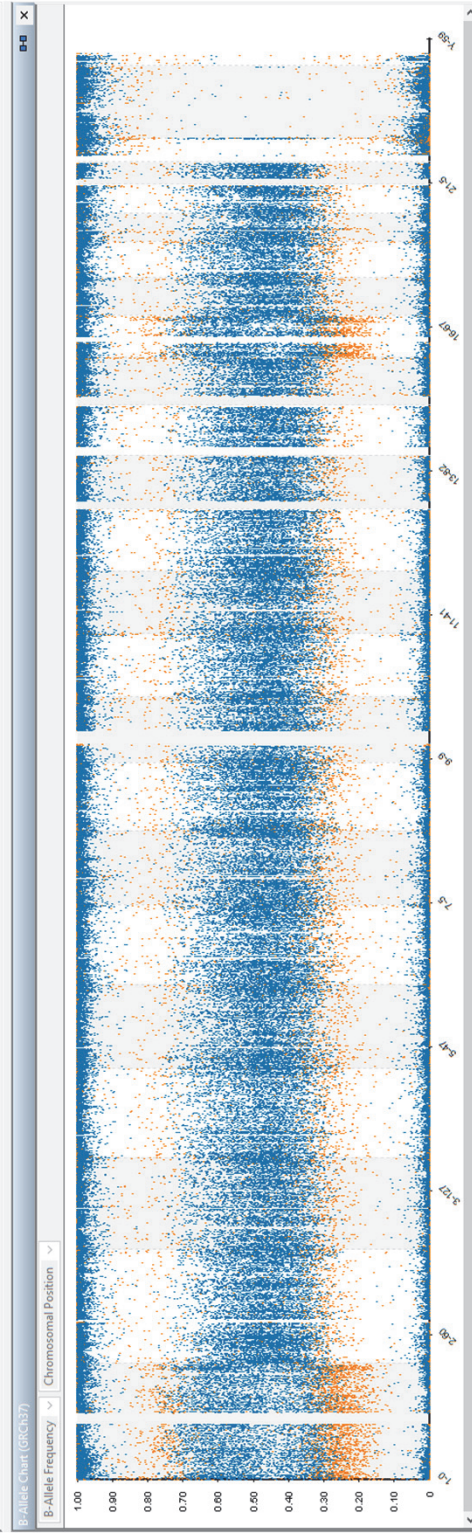

b

Supplement: Supplementary file 1 [file genes-16-00115-s001.zip › Figure S1_250120.pdf]

## Slide 1
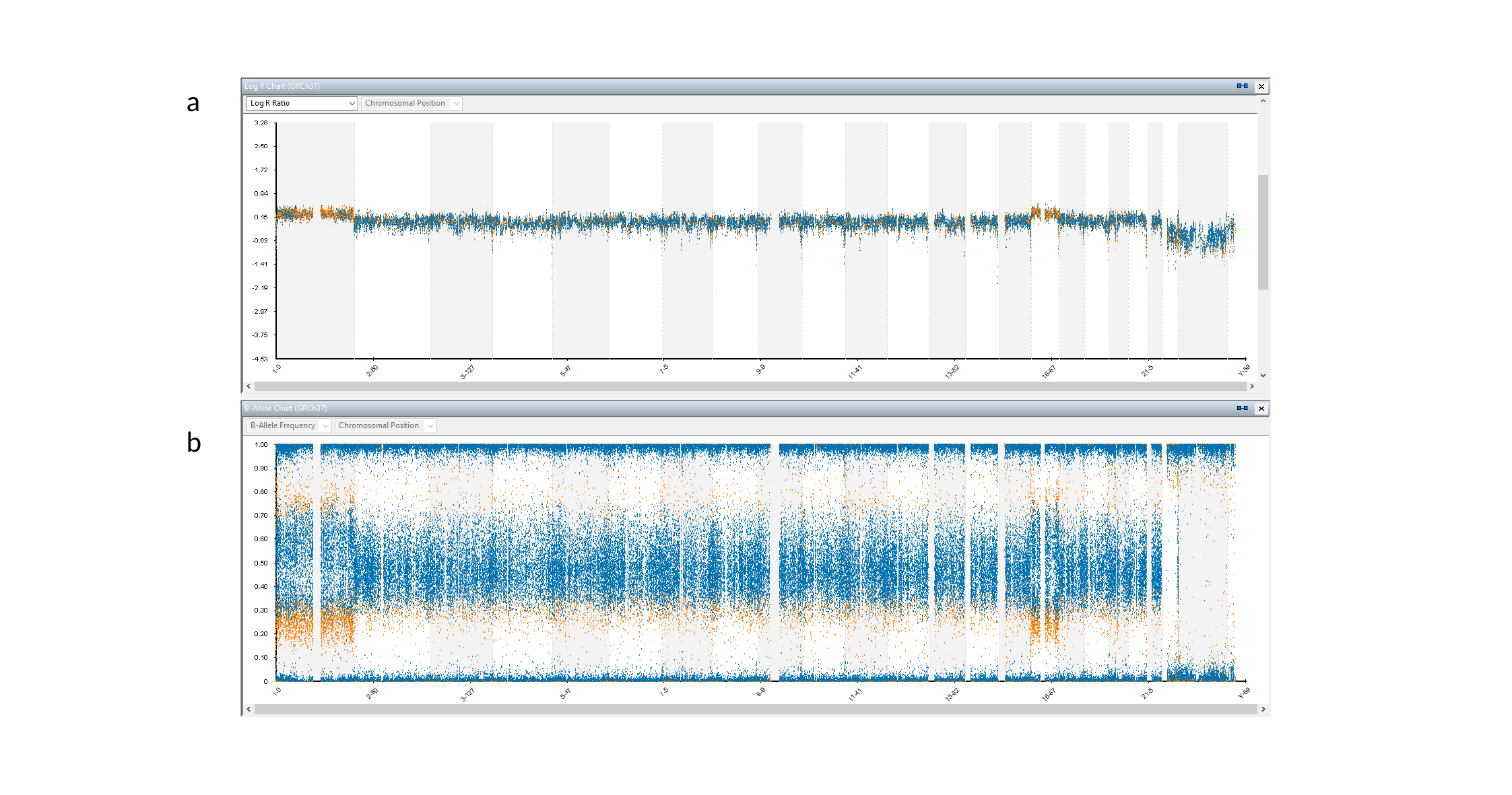

a
b

Supplement: Supplementary file 1 [file genes-16-00115-s001.zip › Figure S1_250120.pptx]

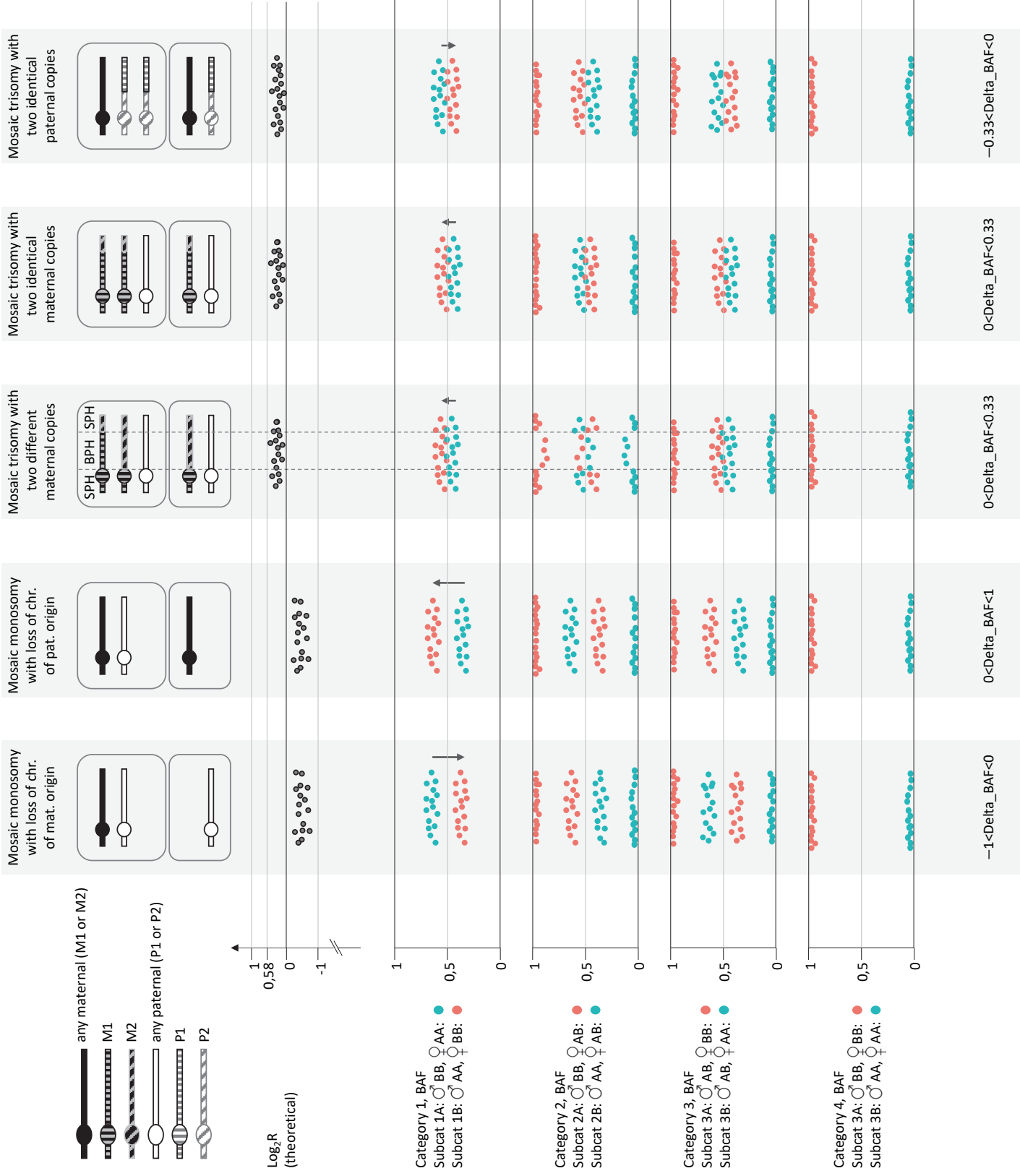

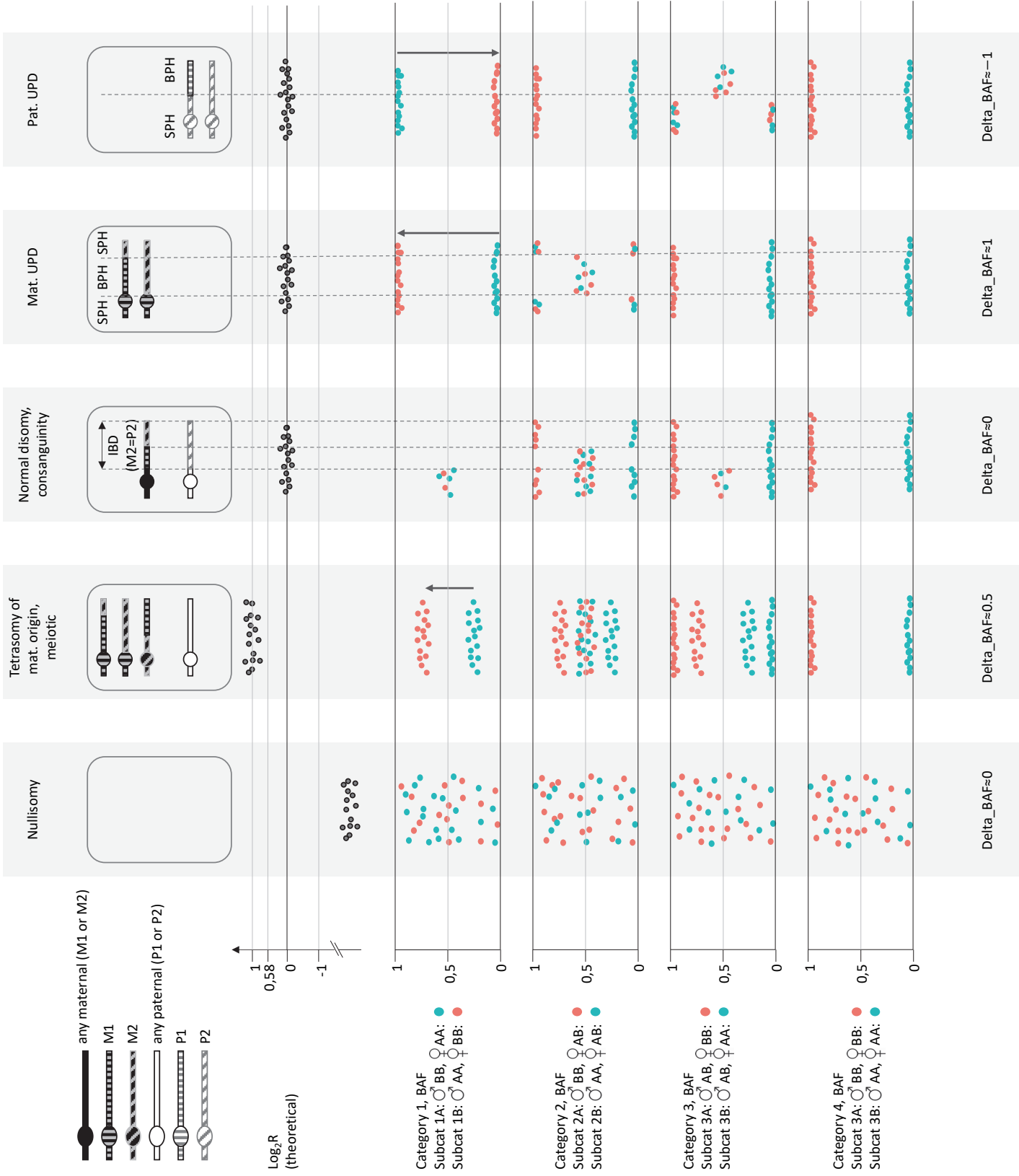

Supplement: Supplementary file 1 [file genes-16-00115-s001.zip › Figure S2_250120.pdf]

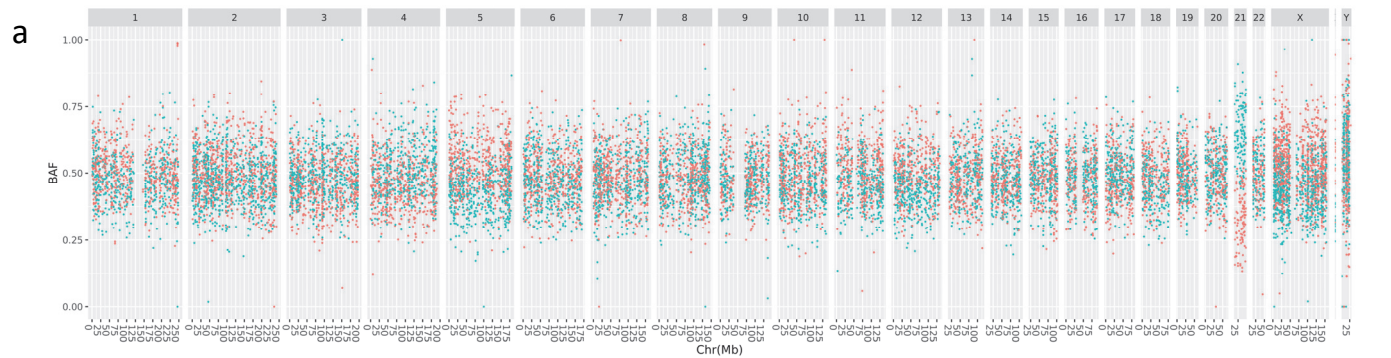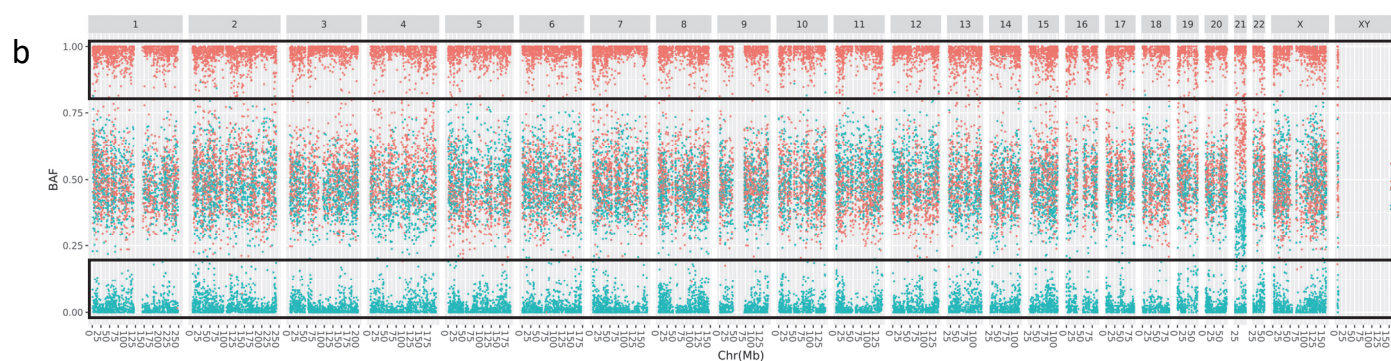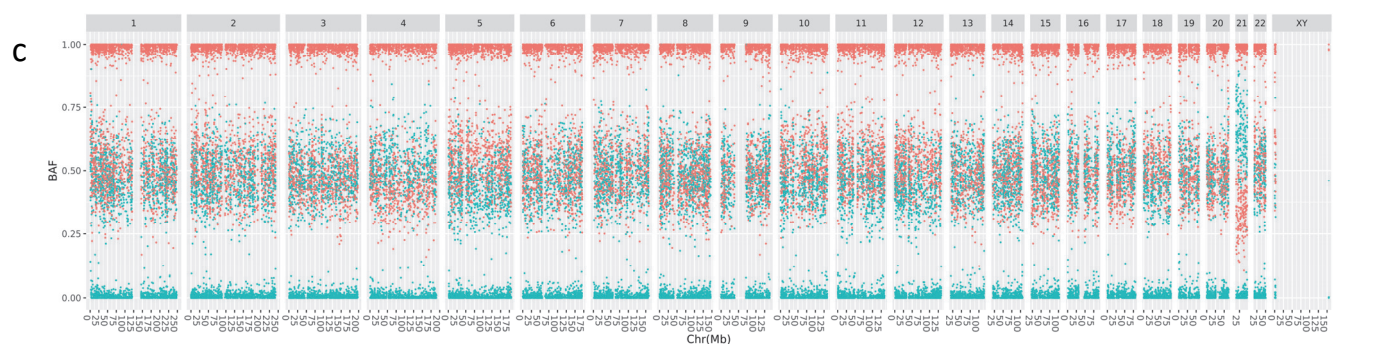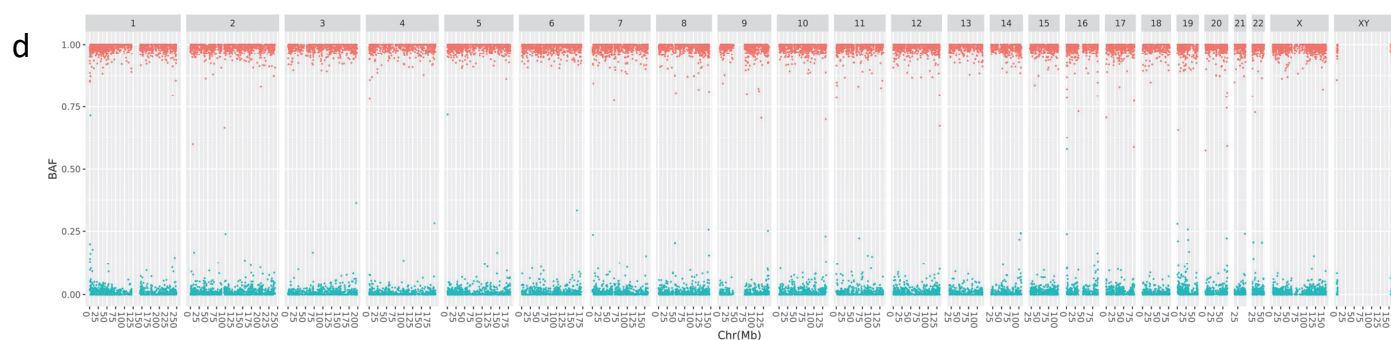

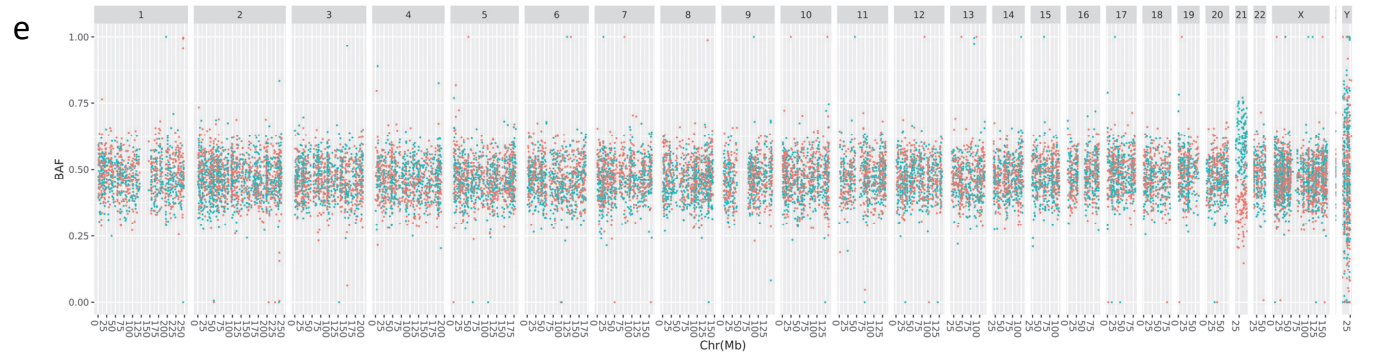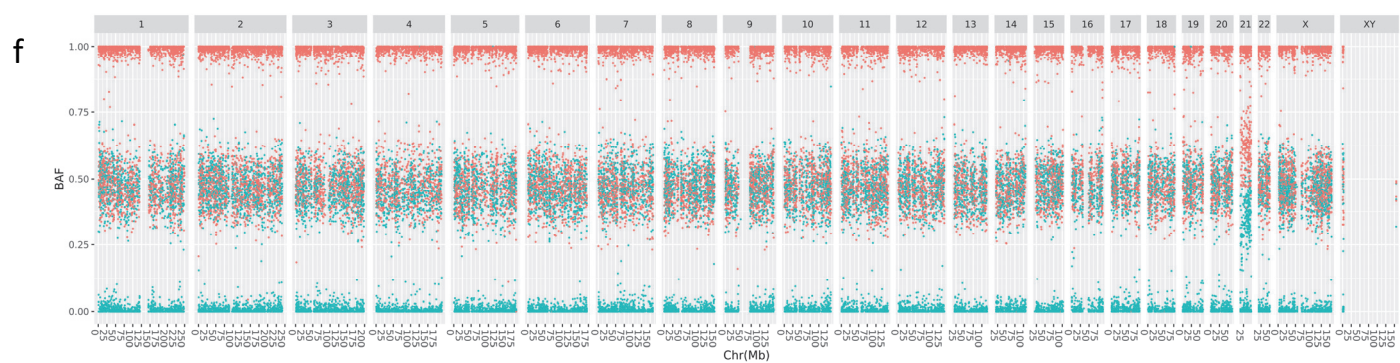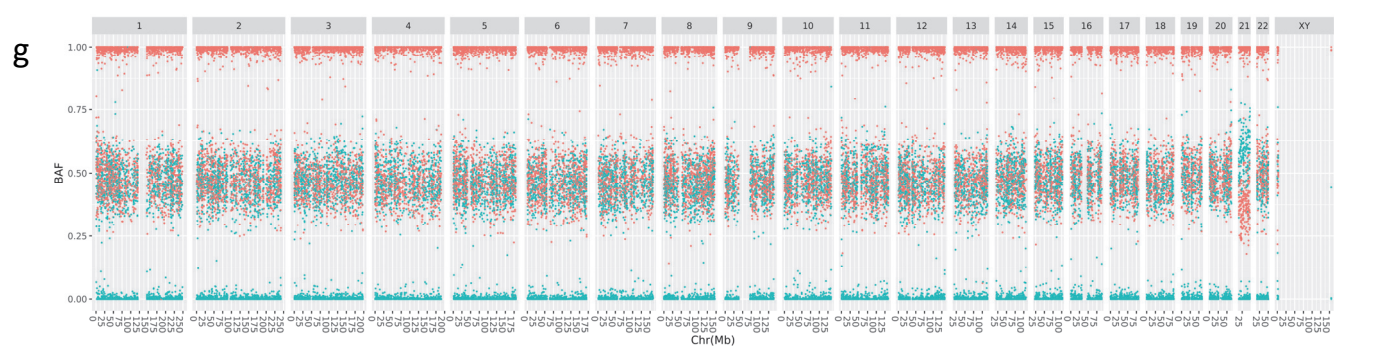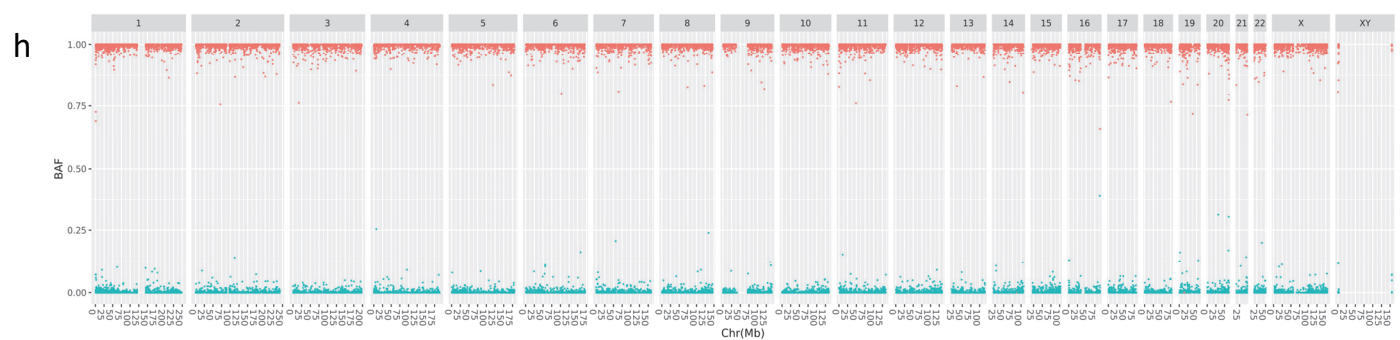

Supplement: Supplementary file 1 [file genes-16-00115-s001.zip › Figure S3_250120.pdf]

## Slide 1
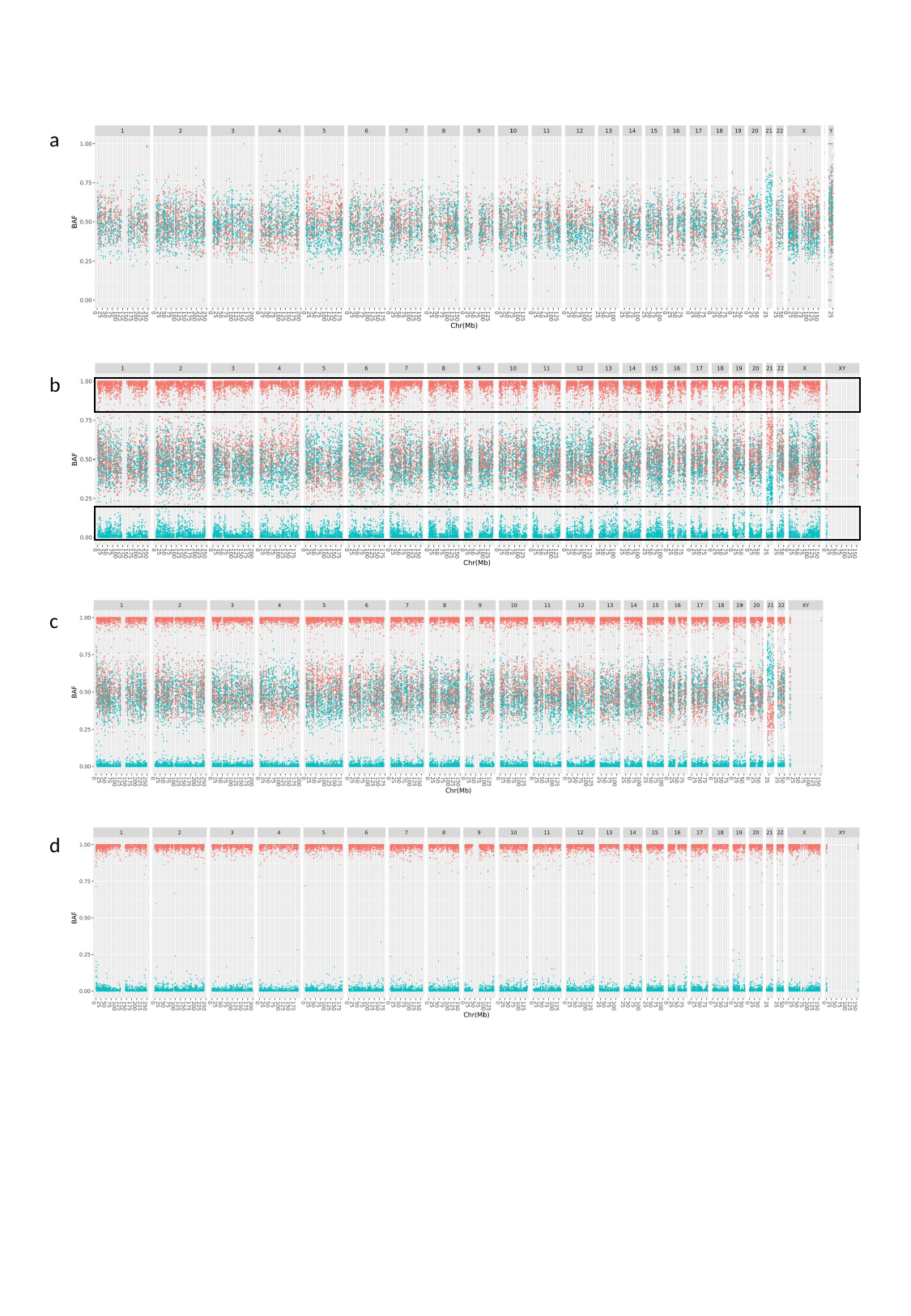

a
b
c
d

## Slide 2
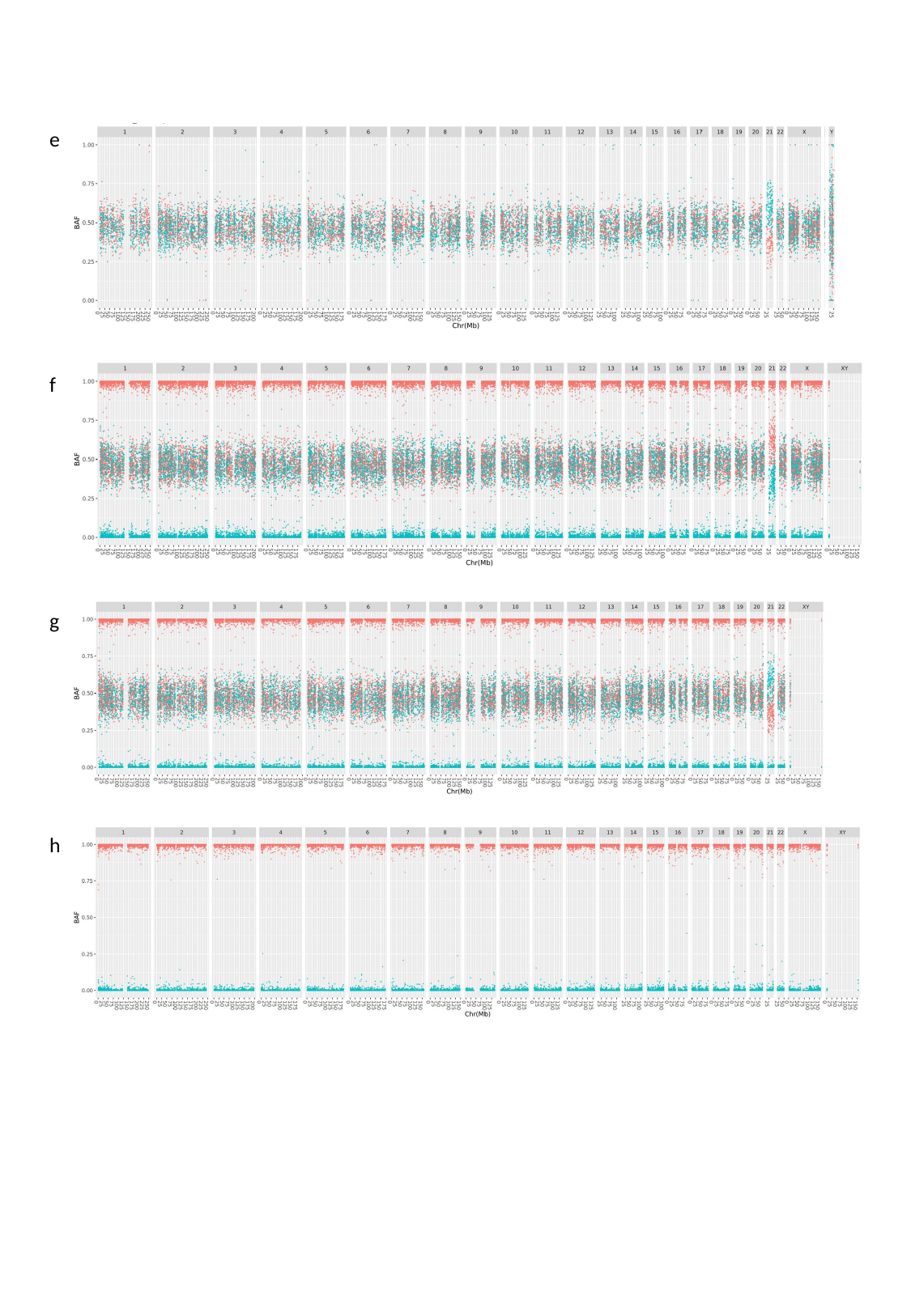

e
f
g
h

Supplement: Supplementary file 1 [file genes-16-00115-s001.zip › Figure S3_250120.pptx]

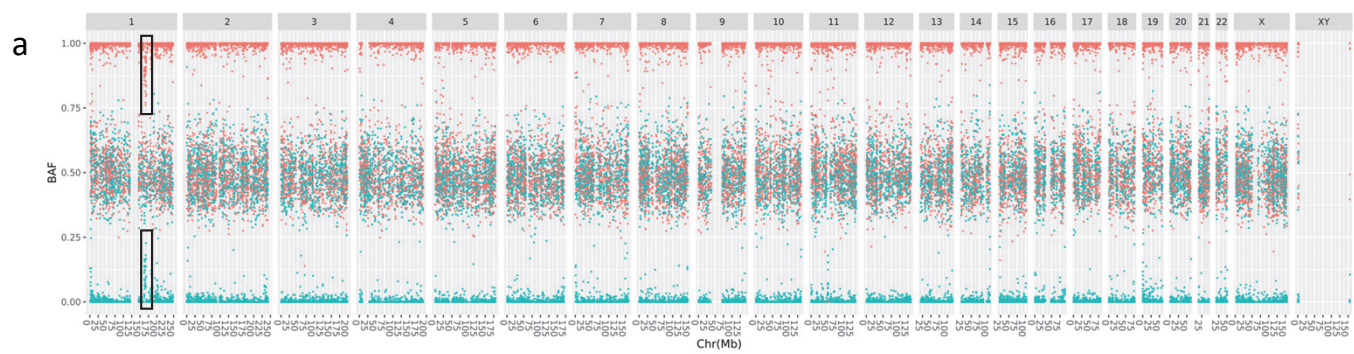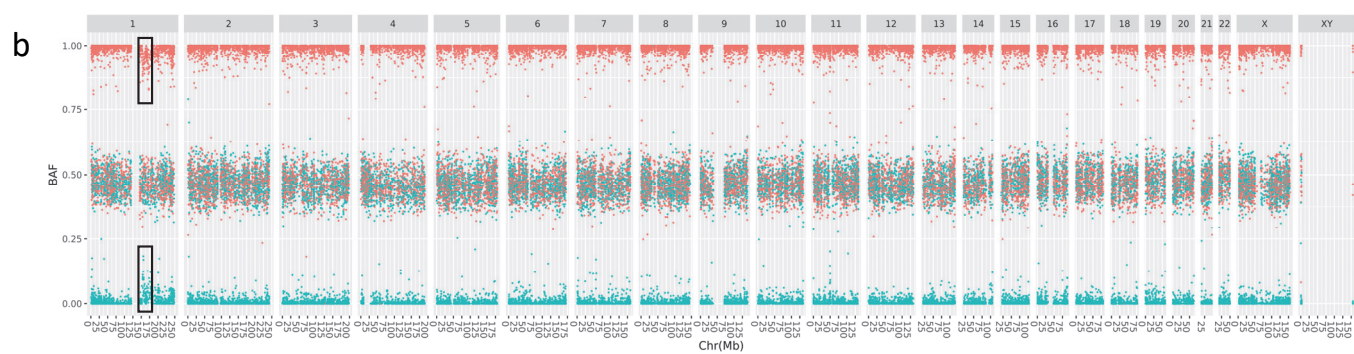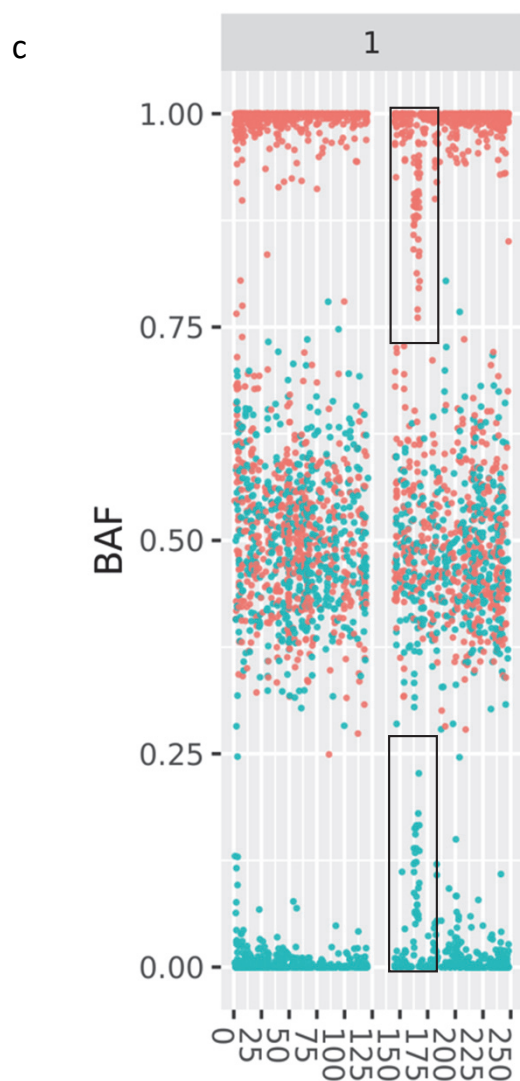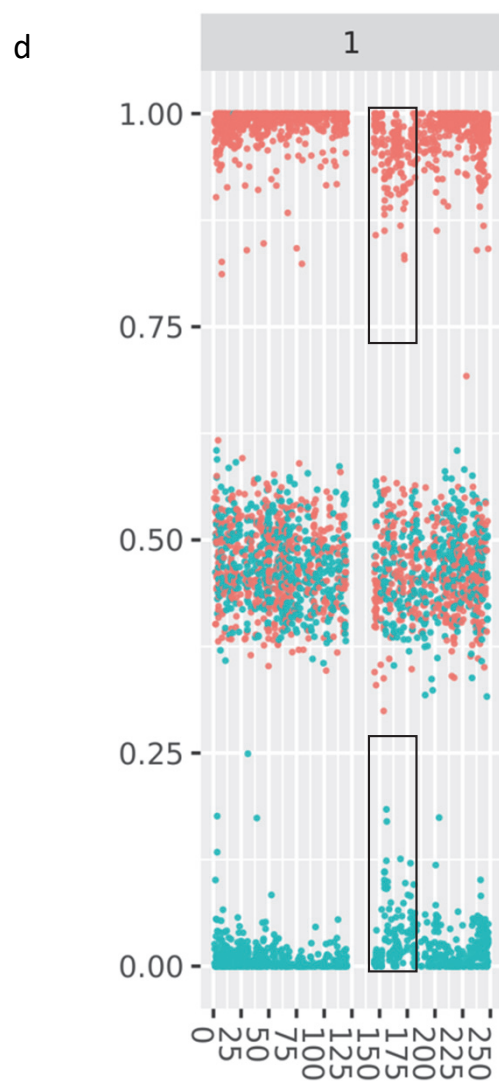

Supplement: Supplementary file 1 [file genes-16-00115-s001.zip › Figure S4_250120.pdf]

## Slide 1
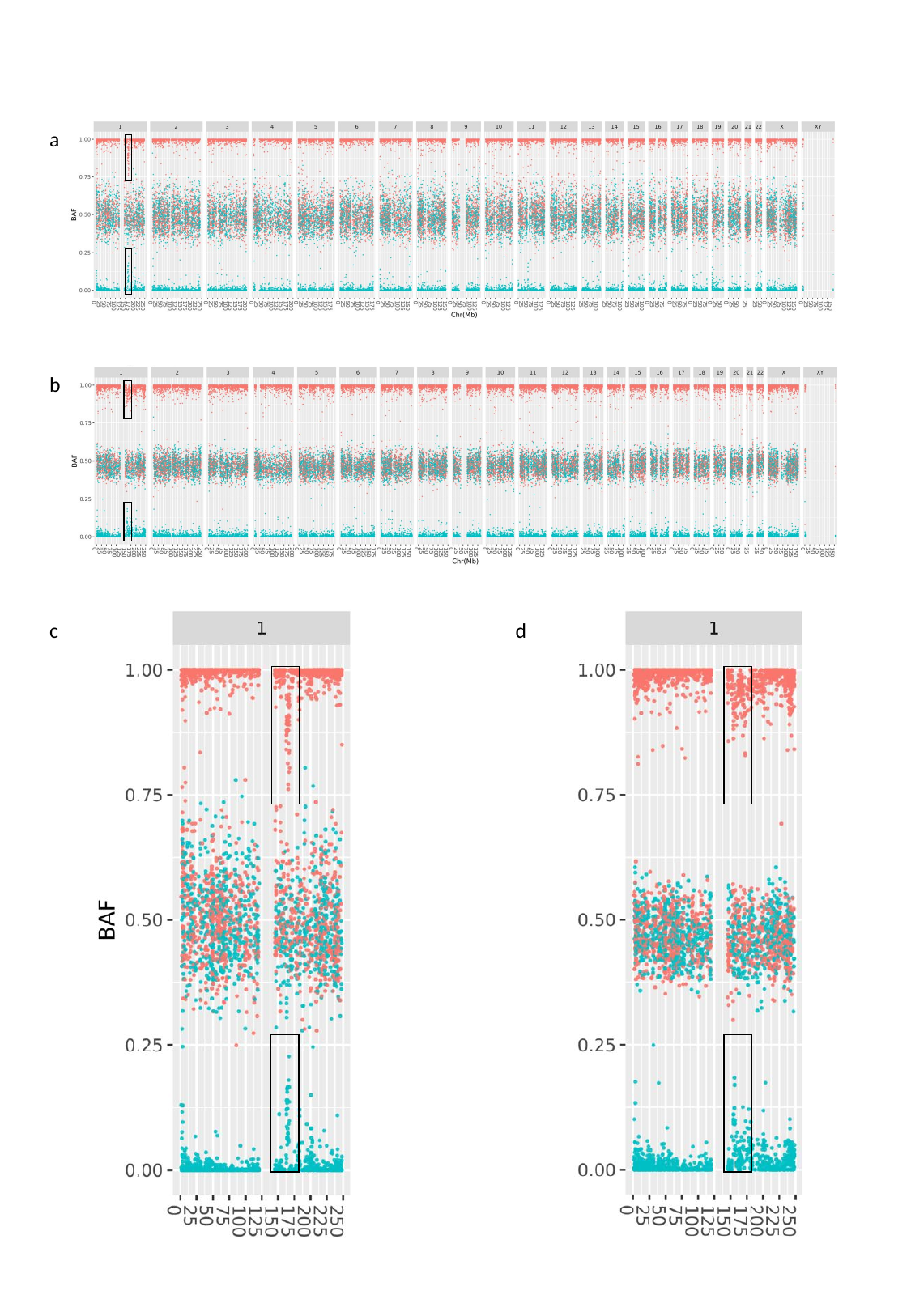

a
b
c
d

Supplement: Supplementary file 1 [file genes-16-00115-s001.zip › Figure S4_250120.pptx]

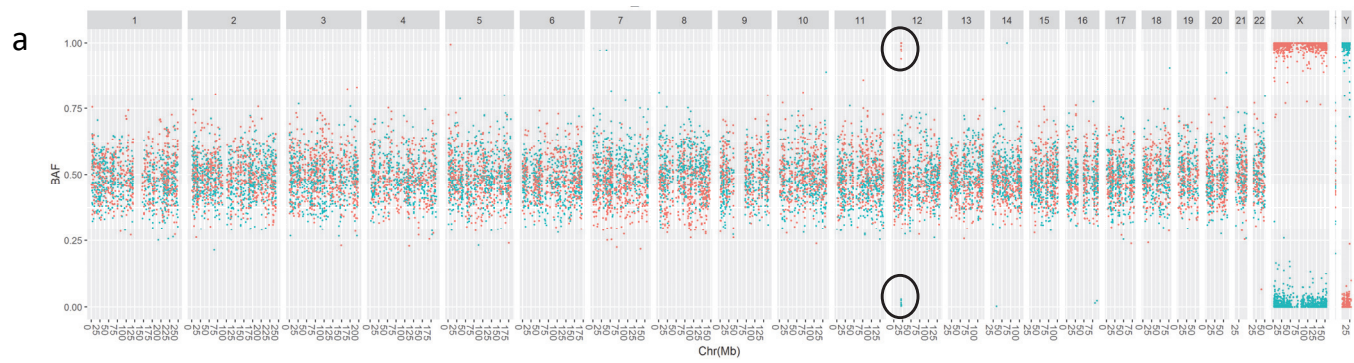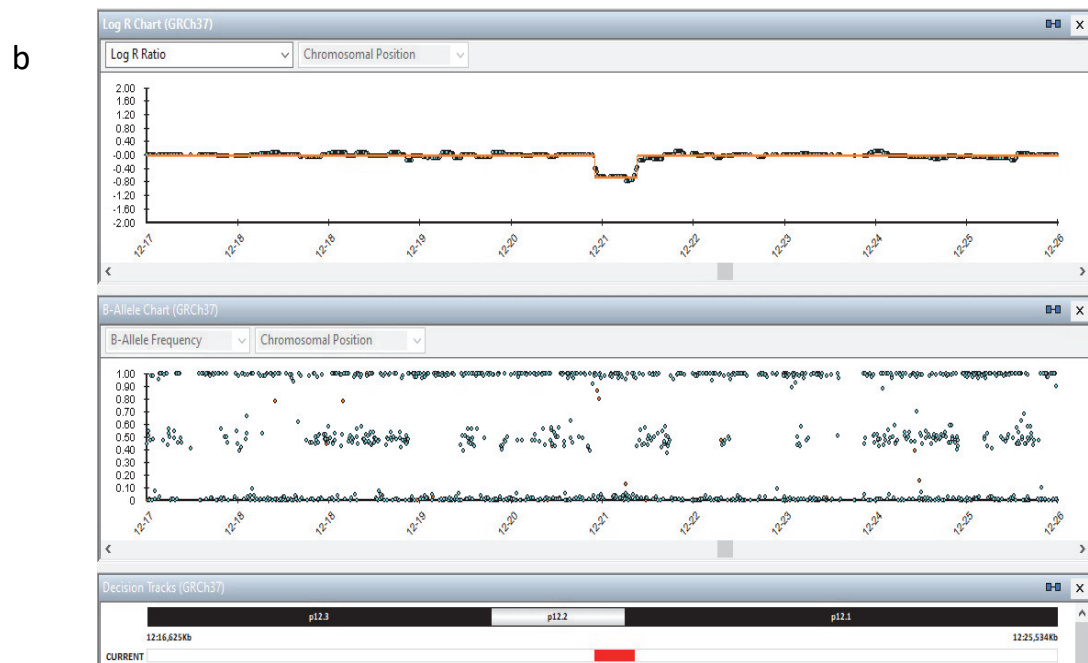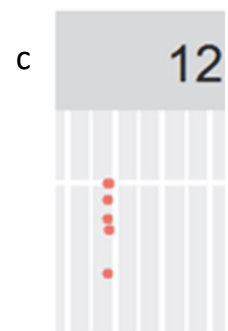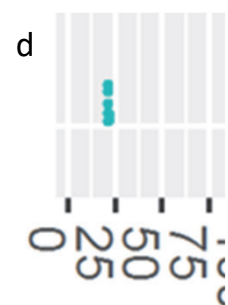

Supplement: Supplementary file 1 [file genes-16-00115-s001.zip › Figure S5_250120.pdf]

## Slide 1
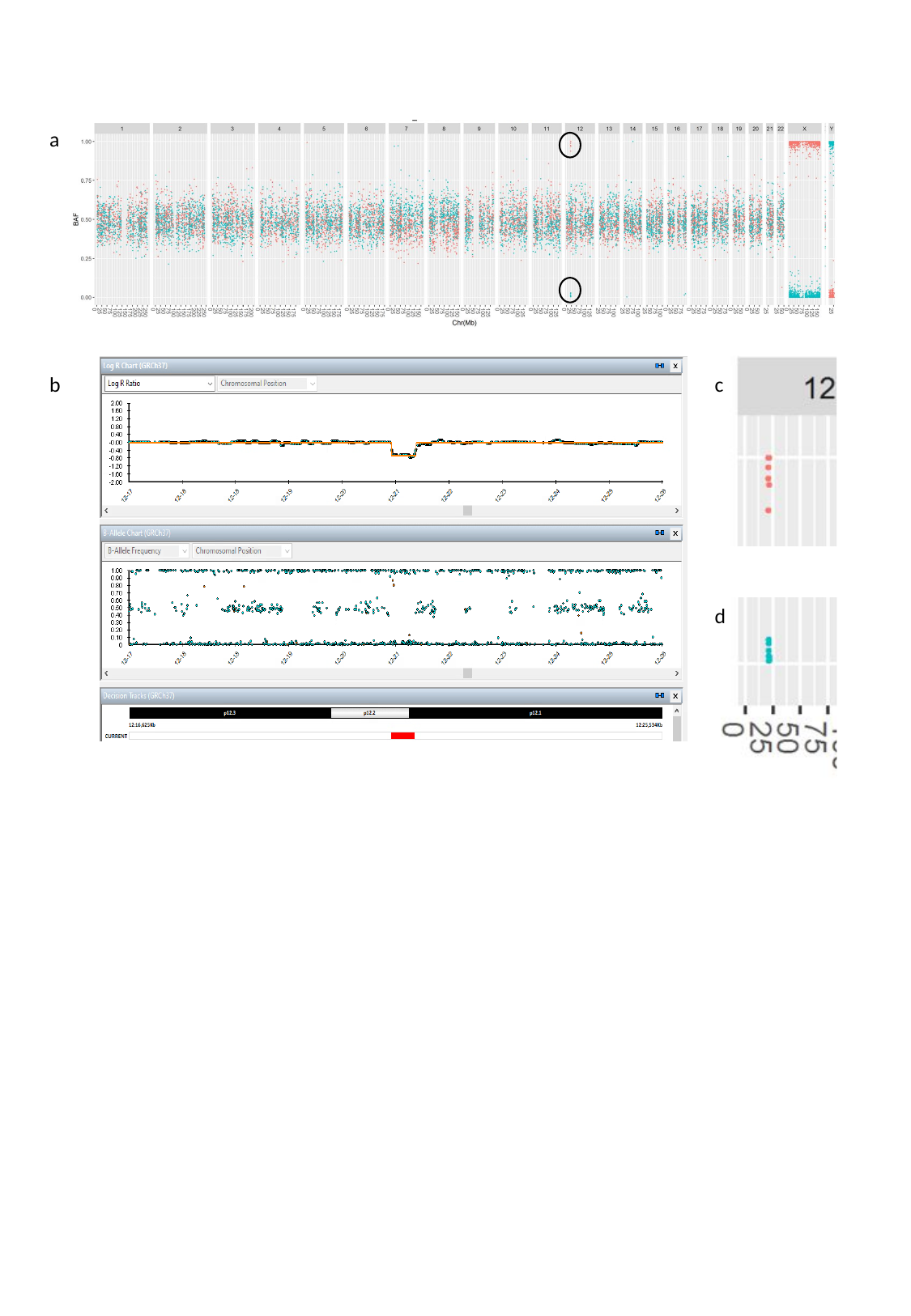

a
b
c
d

Supplement: Supplementary file 1 [file genes-16-00115-s001.zip › Figure S5_250120.pptx]
